# Circulating microRNA-133a in Patients With Arterial Hypertension, Hypertensive Heart Disease, and Left Ventricular Diastolic Dysfunction

**DOI:** 10.3389/fcvm.2020.00104

**Published:** 2020-07-07

**Authors:** Sergiy M. Koval, Iryna O. Snihurska, Kostiantyn O. Yushko, Olga V. Mysnychenko, Marina Yu. Penkova, Olga M. Lytvynova, Alexander E. Berezin, Vadym S. Lytvynov

**Affiliations:** ^1^Department of Arterial Hypertension and Prevention of Its Complications, Government Institution “L.T. Malaya Therapy National Institute of the National Academy of Medical Science of Ukraine, ” Kharkiv, Ukraine; ^2^Department of Laboratory Diagnostics, National University of Pharmacy, Kharkiv, Ukraine; ^3^Internal Medicine Department, Zaporizhia State Medical University, Zaporizhzhia, Ukraine; ^4^Department of Therapy, Rheumatology and Clinical Pharmacology, Kharkiv Medical Academy of Postgraduate Education (KhMAPE), Kharkiv, Ukraine

**Keywords:** arterial hypertension, hypertensive heart disease, microRNA-133a, left ventricular diastolic dysfunction, epigenetics

## Abstract

**Aim:** The aim of the work was to study the circulating microRNA-133a levels in blood plasma of patients with arterial hypertension (AH), hypertensive heart disease (HHD), and left ventricular (LV) diastolic dysfunction (DD).

**Materials and Methods:** A total of 48 patients with grade 2–3 AH and HHD at the age of 52.23 ± 7.26 (23 patients had LV DD [main group] and 25 patients had normal LV diastolic function [comparison group]) and 21 practically healthy individuals of comparable gender and age were examined. Diagnosis of AH and HHD was carried out according to the 2018 ESC/ESH recommendations. LV DD was determined according to the 2016 ASE/EACVI recommendations. Plasma microRNA-133a level was obtained by polymerase chain reaction using the CFX96 Touch System (BioRad), ≪TaqMan microRNA Assay≫ and ≪TaqMan® Universal PCR Master Mix≫ reagent kits (Thermo Fisher Scientific, USA).

**Results:** We have found that in patients from the main and comparison groups plasma microRNA-133a levels were significantly lower than in practically healthy individuals (0.094 [0.067, 0.147]) and (0.182 [0.102, 0.301]) vs. (0.382 [0.198,0.474]), *p* = 0.002 and *p* = 0.04, respectively. In all this among patients with AH, HHD, and LV DD, plasma microRNA-133a levels were significantly lower than in patients with AH, HHD, and normal diastolic function (*p* = 0.03). In the main and comparison groups there was a statistically significant negative relationship between plasma microRNA-133a level and left ventricular mass index (LVMI) (*R* = −0.40, *p* = 0.003 and *R* = −0.35, *p* = 0.04, respectively).

**Conclusions:** The findings suggest the significant role of decreased microRNA-133a levels in blood plasma of patients with AH in the pathogenesis and development of both HHD and LV DD.

## Introduction

Arterial hypertension (AH) is a major public health problem due to its high prevalence all around the globe. More than one billion adults worldwide have AH with up to 45% of the adult populace being affected with the disease (1). The high prevalence of AH is consistent across all socio-economic and income strata, and the prevalence rises with age, accounting for up to 60% of the population above 60 years of age (1, 2).

Chronically elevated blood pressure (BP) may lead to the development of changes in major organs fed by the circulatory system, such as heart, kidneys, brain, and eyes. These changes are grouped under the term “target organ damage” or “hypertension-mediated organ damage.” Heart damage in hypertension has been defined by leading cardiologists in European Union and the United States as “hypertensive heart disease (HHD)” (3–5). Additionally, early manifestation of HHD is left ventricular (LV) hypertrophy (3, 4). Focusing on the HHD it is important to note that chronically increased LV workload in hypertensive patients can result in LV hypertrophy, impaired LV relaxation, development of LV diastolic dysfunction, left atrial enlargement, an increased risk of arrhythmias, especially atrial fibrillation, and an increased risk of heart failure with preserved ejection fraction and heart failure with reduced ejection fraction (1, 3, 4).

There are various mechanisms described for the development of AH which includes increased salt absorption resulting in volume expansion, an impaired response of the renin-angiotensin-aldosterone system (RAAS), increased activation of the sympathetic nervous system. These changes lead to the development of increased total peripheral resistance and increased afterload which in turn leads to the development of AH (5, 6).

A growing body of evidence supports the observation that AH results from a complex interplay of genetic, epigenetic, and environmental factors. Genetic factors are thought to contribute to ~30–60% of BP variation.

However, known genetic factors explain only 3% of BP variance, underscoring the fact that many genetic variants have yet to be discovered. Moreover, these findings suggest that other factors, such as gene–gene interactions and epigenetics, may play a vital role in the etiology of AH. To date, the results of studying the gene polymorphism of a number of powerful factors that regulate vascular tone, inflammation, hypertrophy and fibrosis of the myocardium and remodeling of the vascular wall have been published (7). At the same time, in addition to genetic variations associated with the primary changes in DNA molecules, an important reason for the development and progression of a number of cardiovascular diseases are epigenetic factors regulating gene expression, which include microRNAs (8, 9).

MicroRNAs are small (≈21 nucleotides) non-coding RNAs that negatively regulate gene expression by binding to the 30-UTR sites in the messenger RNAs of protein-coding genes and downregulate their protein expression. Their critical patho-physiological importance is evidenced by their marked evolutionary conservation. Current estimates suggest that they fine-tune expression of up to 50% of protein-coding genes (10, 11). MicroRNAs are crucial for virtually all cellular processes and are a prerequisite for normal cardiac function (9, 12).

Consequently, anomalous microRNA expression profiles are associated with various cardiovascular conditions such as hypertrophy, fibrosis, heart failure, and arrhythmias.

For many years, our department has been studying the development and progression of HHD and the role of adipokines (13), RAAS components (14), and other factors. A new direction in our department is the study of epigenetics. Earlier, we published the results of the study of microRNA-133a in patients with essential AH with the presence and absence of LV hypertrophy (15). As a continuation of this work, aspects of LV diastolic function were studied in patients with HHD. The aim of the study was to investigate the circulating microRNA-133a levels in the blood plasma of patients with arterial AH, hypertensive heart disease, and left ventricular diastolic dysfunction.

## Materials and Methods

Forty-eight moderate-to-severe non-obese hypertensive patients with HHD aged from 48 to 62 years (27 men and 21 women) at the average age 52.23 ± 7.26 were selected from the entire cohort (n = 275) according to the inclusion and exclusion criteria. Inclusion criteria were uncontrolled AH (systolic and/or diastolic BP levels > 140/90 mm Hg), age >18 years, and with written informed consent to participate in the study. Non-inclusion criteria were acute coronary syndrome, acute myocardial infarction, heart failure, atrial fibrillation, type 2 diabetes mellitus, obese, angina pectoris, severe chronic renal failure, acute and chronic inflammatory diseases, severe liver insufficiency, chronic obstructive pulmonary disease, bronchial asthma, pregnancy, malignancy, and disability to know the reason of informed consent.

According to design of the study we separated all patients into two groups: main group, patients with HHD and LV DD (*n* = 23), and comparison group, patients with HHD and normal diastolic function of the LV (*n* = 25). Control group in the present study came from our previous investigation (15). The control group consisted of 21 practically healthy individuals of comparable gender and age.

## Ethical Declaration

The study was approved by the local Ethical Committee (Government Institution “L.T. Malaya Therapy National Institute of the National Academy of Medical Science of Ukraine,” date of approval was 19.01.2017). All patients have given their voluntary informed consent to participate in the study.

## Determination of AH

AH was diagnosed if systolic blood pressure (BP) was >140 mm Hg, and/or diastolic BP > 90 mm Hg according to European guideline on diagnostics and treatment of arterial hypertension (2018) (1), or a self-reported history of AH, and/or the use of anti-hypertensive medications.

## Determination of Dyslipidemia

Dyslipidemia was diagnosed if total cholesterol level was above 5.2 mmol/L, and/or low-density lipoprotein cholesterol (LDL) level was above 3.0 mmol/L, and/or triglyceride level was above 1.7 mmol/L according to the European Cardiology Society dyslipidemia guideline (2016) (16) or use of lipidlowering medication.

## Anthropometric Measurements

Anthropometric measurements [weight, height, body mass, body mass index [BMI], waist circumference, and waist-to-hip ratio] were made using standard procedures (17). Height and weight were measured by professional health staff, with the participants standing without shoes and heavy outer garments with a wall-mounted stadiometer (OMRON, Japan). BMI was calculated as weight (kg) divided by height squared (m^2^). Waist circumference was measured at the level midway between the lower rib margin and the iliac crest, with participants in a standing position without heavy outer garments, with emptied pockets, and breathing out gently. Hip circumference was recorded as the maximum circumference over the buttocks.

## BP Measure

Office BP was measured with the conventional method using a sphygmomanometer (Microlife BP AG 1-10, Hungary).

## ECG Recording

Standard 12-lead electrocardiography was performed at rest according to the conventional method with a three-channel FX-326U ECG recorder (Fukuda, Japan).

## Echocardiography

Echocardiography was conducted in M- and B-modes with a 2.5 MHz phased probe using a medical diagnostic ultrasound complex SSD 280 LS (Aloka, Japan). The LV mass and the LV mass index were calculated by the formula of the American Society of Echocardiography. LV hypertrophy was diagnosed when LV mass index increased to more than 115 g/m^2^ in men and 95 g/m^2^ for women (1).

LV DD was determined according to the 2016 ASE/EACVI recommendations (18). Diagnostic criteria for LV DD were septal e' <7 cm/s, lateral e' <10 cm/s, average E/e' ratio >14, left atrium volume index >34 mL/m^2^, and peak TR velocity >2.8 m/s.

## Calculation of Estimated Glomerular Filtration Rate (EGFR)

eGFR was calculated using CKD-EPI formula (19).

## Blood Sampling

Blood samples were drawn immediately before study entry. Blood serum was initially frozen and stored at −70 to −80°C until testing in the laboratory of immune-chemical and molecular-genetic researches of Government Institution “L.T. Malaya Therapy National Institute of the National Academy of Medical Science of Ukraine.” Blood plasma was isolated within 30 min after centrifugation of blood sample and then frozen at −70 to−80°C and stored in plastic tubes also until being shipped to the laboratory of Institute.

## Biomarker Assay

We determined the levels of fasting plasma glucose, serum urea, creatinine, and uric acid by enzymatic method using Humareazer 2000 analyzer (HUMAN GmbH, Germany). Total cholesterol (TC), low density lipoprotein (LDL) cholesterol, high density lipoprotein (HDL) cholesterol, and triglyceride levels (TG) were measured by direct method on Humareazer 2106 analyzer (HUMAN GmbH, Germany). Serum levels of the N-terminal B type natriuretic peptide (NT-proBNP) (pg/mL) were measured by the enzyme-linked immunosorbent assay (ELISA) with commercial the Elecsys proBNP assay kit manufactured by (Roche Molecular Systems, Inc., Swiss) using Roche Diagnostics Cobas Fara Immunoassay Analyzer (Roche Inc., Swiss).

## MicroRNA-133a Determination

MicroRNA was isolated from 300 μl of plasma using “NucleoSpin miRNA Plasma” kit (Macherey-Nagel, Germany). The miR concentration was determined using “Qubit 3” (Life Technologies, USA) using the “Qubit™ microRNA” (Thermo Fisher Scientific) reagent kit. Reverse transcription was performed using the “TaqMan MicroRNA Reverse Transcription Kit” (Applied Biosystems, USA) and a specific loop primer Hsa-miR-133a (assay ID 002246, Applied Biosystems, USA). Analysis of the microRNA level was performed by real-time polymerase chain reaction (PCR) using the “CFX96 Touch” (BioRad) detection system and the reagent kits for monitoring and analyzing miR expression “TaqMan microRNA Assay” and “TaqMan® Universal PCR Master Mix” (Thermo Fisher Scientific, USA) in accordance with the manufacturer's instructions. Small nuclear RNA U6 (U6 snRNA assay ID 001973, Applied Biosystems, USA) was used as an endogenous control for reverse transcription and amplification. Analysis and calculation of the relative normalized microRNA level was performed using CFX Manager Software (BioRad).

## Statistics

Statistical analysis of the obtained results was performed in the SPSS system for Windows, Version 22 (SPSS Inc., Chicago, IL, USA). The distribution of variables normality was tested with the Kolmogorov-Smirnov test. The data were presented as mean (M) and standard deviation (SD) for normal data distribution or median and interquartile range [Me [25%, 75%]] for abnormal data distribution. To compare the main parameters of patient cohorts, a two-tailed Student *t*-test or Mann-Whitney *U*-test were used. To compare categorical variables between cohorts, the Chi2 test (χ2) and Fisher F exact test were performed. To determine comparisons between three variables we used One-way ANOVA (ANalysis Of VAriance) with *post-hoc* Tukey Honestly Significant Difference. For correlation analysis used Spearmen test. Differences were considered statistically significant at *p* < 0.05.

## Results and Their Discussion

The characteristics of the entire patient study population and both investigated groups are reported in Table 1. The patients of the main and comparison groups didn't differ in such basic characteristics as age, gender composition, severity of AH, the blood levels of lipids, glucose, uric acid, creatinine, diastolic BP. In patients with HHD and LV DD the levels of systolic BP and pulse BP, LV mass index, left atrium size, and E/e' ratio were significantly higher than in patients with HHD and normal LV diastolic function (Table 1). The serum level of NT-proBNP was significantly higher in the main group compared with the control. However, in the main and control groups, the NT-proBNP levels did not exceed 125 pg/mL, which indicated the absence of heart failure in the examined patients (Table 1).

**Table 1:** Basic characteristics of patient study population.

**Parameters**	**Control group (*n* = 21)**	**Entire group (*****n*** **=** **48)**		**Main group (*****n*** **=** **23)**		**Comparison group (*****n*** **=** **25)**		***P*-value between main and comparison group**
		**Parameters**	***p***	**Parameters**	***p***	**Parameters**	***p***	
Age, years	44.14 ± 3.17	52.23 ± 7.26	0.922	53.19 ± 7.21	0.927	51.41 ± 7.29	0.944	0.936
Male, *n* (%)	12 (57%)	27 (56%)	0.912	13(57%)	0.966	14(56%)	0.954	0.912
Female, *n* (%)	9 (43%)	21 (44%)	0.90	10(44%)	0.90	11 (44%)	0.92	0.908
Moderate AH (grade 2 hypertension), *n* (%)	-	21 (44%)	0.0001	9 (39%)	0.0001	12 (48%)	0.0001	0.486
Severe AH (grade 3 hypertension), *n* (%)	-	27 (56%)	0.0001	14(61%)	0.0001	13 (52%)	0.0001	0.423
Systolic BP, mmHg	117.2 ± 10.1	178.3 ± 46.1	0.001	185.7 ± 51.6	0.002	172.6 ± 44.5	0.006	0.004
Diastolic BP, mmHg	77.6 ± 10.3	106.8 ± 31.3	0.006	104.2 ± 29.6	0.03	109.4 ± 32.1	0.041	0.088
Pulse BP, mmHg	43.5 ± 11.1	71.5 ± 22.4	0.03	81.5 ± 24.7	0.02	63.2 ± 20.1	0.043	0.0345
Heart rate, b.p.m.	65.6 ± 8.4	76.4 ± 21.3	0.04	77.3 ± 20.7	0.047	75.6 ± 22.3	0.05	0.446
Dyslipidaemia, *n* (%)	-	37(77%)	0.0001	18 (78%)	0.0001	19 (76%)	0.0001	0.512
Fasting plasma glucose, mmol/L	4.9 ± 1.3	5.4 ± 1.3	0,664	5.3 ± 1.2	0.563	5.5 ± 1.4	0,674	0.844
Serum creatinine, μmol/L	68.5 ± 12.6	88.4 ± 20.1	0.033	90.1 ± 18.6	0.042	87.3 ± 21.4	0.046	0.366
SUA, μmol/L	225.2 ± 37.4	372.6 ± 77.5	0.025	367.9 ± 72.3	0.044	375.6 ± 79.1	0.042	0.412
eGFR, ml/min/1.73 m^2^	94.8 ± 19.9	78.7 ± 18.7	0.041	81.2 ± 19.3	0.047	75.5 ± 17.6	0.028	0.349
Total cholesterol, mmol/L	4.5 ± 0.84	5.92 ± 1.19	0.042	5.87 ± 1.17	0.046	5.95 ± 1.13	0.044	0.426
LDL cholesterol, mmol/L	2.3 ± 0.49	3.94 ± 0.82	0.042	3.89 ± 0.79	0.051	3.96 ± 0.83	0.048	0.455
HDL cholesterol, mmol/L (male)	1.3 ± 0.17	0.95 ± 0.27	0.721	0.96 ± 0.26	0.824	0.94 ± 0.28	0.92	0.166
HDL cholesterol, mmol/L (female)	1.5 ± 0.19	1.13 ± 0,26	0.684	1.12 ± 0,25	0.882	1.14 ± 0.27	0.816	0.147
Triglycerides, mmol/L	1.4 ± 0.25	1.8 ± 0.38	0.512	1.77 ± 0.37	0.518	1.84 ± 0.41	0,684	0.673
NT-proBNP, pg/mL	38.42 [27.15,45.79]	66.55[40.05,86.22]	0.006	75.93 [52.39,95.54]	0.009	57.67 [34.58, 77.45]	0.022	0.0438
LV mass index, g/m^2^ (male)	76 ± 20.3	124.3 ± 31.4	0.01	129.3 ± 33.5	0.005	118.8 ± 29.3	0.02	0.0299
LV mass index, g/m^2^ (female)	68 ± 18.4	105.7 ± 28.8	0.019	111.5 ± 29.7	0.017	99.2 ± 27.4	0.03	0.0447
Ejection fraction, %	68.3 ± 7.2	61.3 ± 14.3	0.422	59.1 ± 12.7	0.612	62.4 ± 14.2	0.414	0.296
Left atrial size, ml/m^2^ (male)	16.6 ± 3.9	18.6 ± 4.3	0.022	19.3 ± 4.7	0.032	17.9 ± 4.1	0.028	0.0455
Left atrial size, ml/m^2^ (female)	13.9 ± 2.2	16.7 ± 3.9	0.03	17.4 ± 4.1	0.026	16.0 ± 3.7	0.027	0.0385
E/e′ (averaged) ratio	8.2 ± 2.2	12.8 ± 3.2	0.004	13.9 ± 3.5	0.018	11.5 ± 3.1	0.019	0.044

*AH, arterial hypertension; BP, blood pressure; SUA, serum uric acid; LV, left ventricular; eGFR, estimated glomerular filtration rate; HDL, high density lipoprotein; LDL, low-density lipoprotein; NT-proBNP, N-terminal B type natriuretic peptide*.

*ANOVA was used to determine statistical comparisons among 3 groups (p)*.

**Figure 1:**
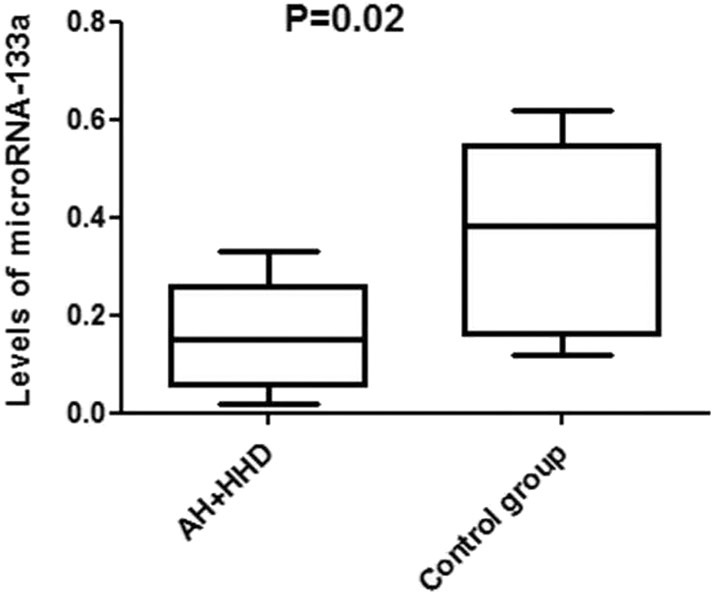
Levels of microRNA-133a in blood plasma in patients with AH and HHD and healthy individuals in the control group. AH, arterial hypertension; HHD, hypertension heart disease.

When studying the levels of microRNA-133a in plasma and clinical, hemodynamic, metabolic, and cardiac parameters in AH patients with HHD and DD or without DD, the following results were obtained. It was established that in entire group of patients with HDD the plasma levels of microRNA-133a were significantly lower than in practically healthy individuals in the control group (0.152 [0.092, 0.189]) vs. (0.382 [0.198, 0.474]), *P* = 0.02 (Figure 1).

**Figure 2:**
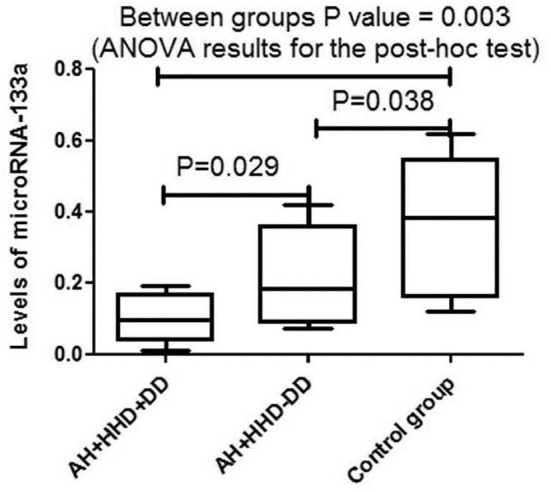
Levels of microRNA-133a in blood plasma in patients with AH and HHD depending on the presence or absence of LV DD. AH, arterial hypertension; HHD, hypertension heart disease; DD, diastolic dysfunction.

In patients from the main and comparison groups microRNA-133a levels were significantly lower than in practically healthy individuals [0.094 [0.067, 0.147], *P* = 0.003] and (0.182 [0.102, 0.301]) vs. [0.382 [0.198, 0.474] *P* = 0.038] (Figure 2). There were significant differences between AH+HHD+DD group and AH+HDD-DD group (*P* = 0.029). The condition studentized range critical values were 0.048; 2.78; 12.2. Tukey HSD pairwise comparisons for variables were the following: HSD-test for AHH+HHD+DD group vs. AH+HHD-DD group 3.12; HSD-test for AHH+HHD+DD group vs. control group = 7.26; HSD-test for AHH+HHD-DD group vs. control group = 4.25.

When analyzing plasma microRNA-133a levels in the main and comparison groups depending on the BP measurements, a significant negative correlation has been established in systolic BP(R = −0.47, *p* = 0.003 and R = −0.42, *p* = 0.004, respectively) and pulse BP(R = −0.45, *p* = 0.03, and *R* = −0.39, *p* = 0.04, respectively) levels, without finding one in the diastolic BP levels and heart rate (Table 2). At the same time in the main and comparison groups there was a statistically significant negative correlation between plasma microRNA-133a level and LV mass index (*R* = −0.40, *p* = 0.003 and *R* = −0.35, *p* = 0.04, respectively) with no significant correlation with ejection fraction, left atrial size, or E/e' (averaged) ratio (Table 2).

**Table 2:** Correlations between the levels of microRNA-133a in blood plasma and clinical, hemodynamic, metabolic, and cardiac parameters in control group and in hypertension patients with HHD and DD or without DD.

**Variables**	**Control group (*****n*** **=** **21)**		**Main group (*****n*** **=** **23)**		**Comparison group (*****n*** **=** **25)**	
	***R***	***p***	***R***	***p***	***R***	***p***
Age, years	−0.005	0.426	−0.09	0.128	−0.11	0.187
Systolic BP, mmHg	−0.06	0.166	−0.47	0.003	−0.42	0.004
Diastolic BP, mmHg	−0.003	0.533	−0.29	0.186	−0.26	0.268
Pulse BP, mmHg	−0.08	0.408	−0.45	0.032	−0.39	0.04
Heart rate, b.p.m.	−0.007	0.712	−0.22	0.187	−0.15	0.411
Fasting plasma glucose, mmol/L	0.03	0.209	0.14	0.225	0.19	0.392
Serum creatinine, μmol/L	0.03	0.224	0.07	0.643	−0.09	0.410
SUA, μmol/L	0.06	0.438	0.11	0.511	−0.08	0.392
eGFR, ml/min/1.73 m^2^	−0.07	0.337	−0.16	0.338	0.11	0.618
Total cholesterol, mmol/L	0.04	0.442	0.18	0.452	0.07	0.376
LDL cholesterol, mmol/L	−0.02	0.407	−0.23	0.448	−0.14	0.447
HDL cholesterol, mmol/L	0.02	0.472	0.12	0.388	0.06	0.342
Triglycerides, mmol/L	−0.008	0.567	−0.08	0.325	−0.16	0.290
NT–proBNP, pg/mL	−0.10	0.299	−0.25	0.311	−0.15	0.281
LV mass index, g/m^2^	−0.15	0.329	−0.40	0.003	−0.35	0.04
Ejection fraction, %	0.10	0.312	0.12	0.356	0.19	0.244
Left atrial size, ml/m^2^	−0.11	0.366	−0.31	0.262	−0.22	0.285
E/e′ (averaged) Ratio	0.12	0.346	0.23	0.416	0.17	0.388

*HHD, hypertension heart disease; DD, diastolic dysfunction; BP, blood pressure; SUA, serum uric acid; LV, left ventricular; eGFR, estimated glomerular filtration rate; HDL, high-density lipoprotein; LDL, low-density lipoprotein; NS, not significant*.

The results obtained are generally consistent with published data on the change in the level of circulating microRNA-133a in the AH and HHD. Thus, in Zhang et al. (20) in patients with essential AH, a decrease in the level of microRNA-133a in serum was found in comparison with the control group of practically healthy individuals (20). Studies of the nature of changes in the level of circulating microRNA-133a in patients with AH from the European population include the work of (12). In this work, a significant decrease in the level of expression of circulating microRNA-133a in mononuclear blood cells in patients with AH was shown in comparison with that in practically healthy individuals. In another work by Kontaraki et al. it was found a significant decrease in the expression level of microRNA-133 as a whole as a family in blood cells in patients with AH in comparison with healthy individuals (21). Moreover, in the works of Kontaraki et al. were shown significant positive correlations of the expression level of microRNA-133 as a whole as a family with 24-h ambulatory BP, mean diastolic BP, and mean pulse pressure (21) and the significant inverse correlation was found between the level of microRNA-133a and LV mass index (*R* = −0.431, *p* < 0.001) (12).

MicroRNA-133a has a critical role in determining cardiomyocyte hypertrophy; its over-expression inhibits hypertrophy whereas its suppression induces hypertrophy both *in vitro* and *in vivo* (22).

According to modern data, the microRNA-133 family is considered as powerful factors that inhibit myocardial hypertrophy and fibrosis, including in response to mechanical overload, in particular pressure overload (22, 23). At the same time, in an experiment on salt-sensitive rats, it was found that high-salt intake in these rats not only leads to an increase in BP but also to suppression of microRNA-133a myocardial expression that initiates fibrosis of animals myocardium (24). The most important function of microRNA-133a is its participation in the development, differentiation of the cardiomyocytes and regulation of the processor of hypertrophy and fibrosis of the myocardium (11, 24). In this regard, the family of microR-133 refers to the so-called “myo-miRs” (10).

Thus, the decrease in microRNA-133a level in plasma in patients with AH and HHD, especially with LV DD, revealed by us indicates the important role of the deficiency of this microRNA in the pathogenesis and development of both HHD and LV DD.

The question of the primary or secondary depression of miRNA-133a in patients with hypertension and LVH and LVDD remains open today. However, the available data indicate the promise of a search for methods to increase miRNA-133a production to inhibit the processes of hypertrophy and fibrosis of the myocard (10, 22).

## Conclusions

We have established a significant decrease in plasma levels of microRNA-133a in patients with grade 2–3 AH and HHD. The lowest level of microRNA-133a in plasma has been detected in patients with the presence of AH, HHD, and LV DD. The findings suggest a significant role of decreased microRNA-133a levels in blood plasma of patients with AH in the pathogenesis and development of both HHD and LV DD.

## Data Availability Statement

All datasets generated for this study are included in the article/supplementary material.

## Ethics Statement

The study was approved by the local Ethical Committee (Government Institution L.T. Malaya Therapy National Institute of the National Academy of Medical Science of Ukraine, date of approval was 19.01.2017). All patients have given their voluntary informed consent to participate in the study.

## Author Contributions

All persons who meet authorship criteria are listed as authors, and all authors certify that they have participated sufficiently in the work to take public responsibility for the content, including participation in the concept, design, analysis, writing, or revision of the manuscript. Furthermore, each author certifies that this material or similar material has not been and will not be submitted to or published in any other publication before its appearance in the Frontiers in Cardiovascular Medicine.

## Conflict of Interest

The authors declare that the research was conducted in the absence of any commercial or financial relationships that could be construed as a potential conflict of interest.
